# Functional Testing of an Inhalable Nanoparticle Based Influenza Vaccine Using a Human Precision Cut Lung Slice Technique

**DOI:** 10.1371/journal.pone.0071728

**Published:** 2013-08-13

**Authors:** Vanessa Neuhaus, Katharina Schwarz, Anna Klee, Sophie Seehase, Christine Förster, Olaf Pfennig, Danny Jonigk, Hans-Gerd Fieguth, Wolfgang Koch, Gregor Warnecke, Vidadi Yusibov, Katherina Sewald, Armin Braun

**Affiliations:** 1 Fraunhofer Institute for Toxicology and Experimental Medicine, Hannover, Germany; 2 Research Center Borstel, Leibniz Center for Medicine and Biosciences Airway Reserach Center North (ARCN), Borstel, Germany; 3 KRH Klinikum Oststadt-Heidehaus, Hannover, Germany; 4 Institute for Pathology, Hanover Medical School, Hannover, Germany; 5 Division of Cardiac, Thoracic, Transplantation, and Vascular Surgery, Hanover Medical School, Hannover, Germany; 6 Fraunhofer USA Center for Molecular Biotechnology, Newark, Delaware, United States of America; 7 Institute of Immunology, Hanover Medical School, Hannover, Germany; 8 Biomedical Research in Endstage and Obstructive Lung Disease Hannover (BREATH), Member of the German Centre for Lung Research (DZL), Hannover, Germany; 9 German Center for Lung Research (DZL), Hannover, Germany; Instituto Butantan, Brazil

## Abstract

Annual outbreaks of influenza infections, caused by new influenza virus subtypes and high incidences of zoonosis, make seasonal influenza one of the most unpredictable and serious health threats worldwide. Currently available vaccines, though the main prevention strategy, can neither efficiently be adapted to new circulating virus subtypes nor provide high amounts to meet the global demand fast enough. New influenza vaccines quickly adapted to current virus strains are needed. In the present study we investigated the local toxicity and capacity of a new inhalable influenza vaccine to induce an antigen-specific recall response at the site of virus entry in human precision-cut lung slices (PCLS). This new vaccine combines recombinant H1N1 influenza hemagglutinin (HAC1), produced in tobacco plants, and a silica nanoparticle (NP)-based drug delivery system. We found no local cellular toxicity of the vaccine within applicable concentrations. However higher concentrations of NP (≥10^3^ µg/ml) dose-dependently decreased viability of human PCLS. Furthermore NP, not the protein, provoked a dose-dependent induction of TNF-α and IL-1β, indicating adjuvant properties of silica. In contrast, we found an antigen-specific induction of the T cell proliferation and differentiation cytokine, IL-2, compared to baseline level (152±49 pg/mg vs. 22±5 pg/mg), which could not be seen for the NP alone. Additionally, treatment with 10 µg/ml HAC1 caused a 6-times higher secretion of IFN-γ compared to baseline (602±307 pg/mg vs. 97±51 pg/mg). This antigen-induced IFN-γ secretion was further boosted by the adjuvant effect of silica NP for the formulated vaccine to a 12-fold increase (97±51 pg/mg vs. 1226±535 pg/mg). Thus we were able to show that the plant-produced vaccine induced an adequate innate immune response and re-activated an established antigen-specific T cell response within a non-toxic range in human PCLS at the site of virus entry.

## Introduction

Every year, seasonal influenza occurs as an infectious viral disease of the respiratory tract that is caused by RNA viruses of the Orthomyxoviridae family.

Influenza viruses present serious health threats worldwide, being responsible for annual global epidemics causing several million cases of severe illness and deaths in higher risk groups [1]. Among three types of seasonal influenza viruses (A, B and C), type A influenza virus further subdivides into serotypes classified according to antigenic specificity of their surface glycoproteins, hemagglutinin (HA) and neuraminidase (NA) [2]. In order to prevent seasonal influenza infections, the World Health Organization (WHO) recommends annual vaccination, especially in high-risk groups including infants (<2 years), elderly individuals (>65 years), and people with chronic medical conditions [1].

Seasonal influenza vaccines are based on circulating influenza virus types. Thus, there is a risk of losing vaccination efficacy due to the high mutation rate of the influenza virus [3]. In fact, this reduced protection against mutated virus subtypes became especially apparent during the H1N1 pandemic outbreak in 2009. The high mutation rate of the influenza virus, mainly antigenic changes of HA and NA, enables the virus to escape neutralization by pre-existing antibodies in the host [4], and causes new seasonal virus subtypes [5], [6]. Moreover, a high frequency of interspecies transmission and reassortment of the virus make influenza infections highly unpredictable [7], [8]. In this context, there is only a short time frame for the production of adapted influenza vaccines, from the identification of the present virus strain to its outbreak in the population. This requires a fast production of adapted influenza vaccines. Conventional seasonal influenza vaccines are mostly based on traditional but time-consuming chicken egg-based production processes allowing only little scope for fast adjustments [9]–[11]. Moreover, systemic immunization with adjuvants that have limited efficiency requires high amounts of vaccines to be supplied to the global market. A remedy would be, on one hand, a faster and increased production of vaccines using improved processes. On the other hand, optimization of the application at the site of the virus entry may increase the vaccine efficiency and reduce the vaccine dosage enabling even a small-scale production to provide sufficient amounts of vaccines to the global market.

In the past few years plant-based vaccine production processes were developed to overcome the time-consuming influenza vaccine production during pandemics. The production of recombinant virus proteins, such as HA, in rapidly growing plants may enable a more cost-efficient and faster vaccine production with a higher flexibility to adjust to new virus subtypes than the conventional egg-based production [11]–[13].

Currently, most vaccines are administered by intramuscular injection. The muscle however only contains few macrophages, dendritic cells or lymphocytes and is therefore not considered as an optimal site for antigen presentation and T cell activation, which may lead to relatively weak humoral and cellular immune response [14], [15].

Contrary to that, local mucosal immunization with an adjuvant via the respiratory tract at the site of the virus entry may stimulate local humoral and cell-mediated immune responses [16], [17]. Consequently, mucosally administered adjuvanted influenza vaccines showed increased immunogenic effects with potential to reduce the required vaccine doses to be supplied to the global market [18], [19]. In context of local vaccination, a drug delivery system like micro- or nanoparticles (NP) might be of great benefit in terms of protein stabilization or controlled antigen release [20], [21]. Some NP provided an adjuvant effect, which improved the local immunogenicity of a vaccine without the use of additional adjuvants [22]–[24].

Development of an adequate model which closely resembles the human situation at the port of the virus entry is essential. On one hand, there are well established *in vivo* models using rodents, ferrets, or non-human primates for elucidating the pathogenesis of influenza infections and developing new influenza vaccines [25]. On another hand, there are *in vitro* models focusing on a single cell population to conduct cellular assays [26]. A model that links the simple, artificial *in vitro* and complex *in vivo* situation is the *ex vivo* method of precision-cut lung slices (PCLS). In particular for respiratory vaccination, the PCLS model allows for elucidating cellular mechanisms and interaction within the lung parenchyma. Previous studies at our department showed that human PCLS mimic the human *in vivo* situation in respect to local pulmonary effects on the innate immune system by well-known modulators [27]. Furthermore, Wu and colleagues demonstrated that human PCLS infected with influenza virus developed innate immune responses as determined by cytokine production [28], making this system valuable for testing of experimental vaccines.

In the present study, we investigated the local toxicity of a new inhalable influenza vaccine as well as its potential to recall an immune response at the point of the virus settlement in human PCLS. This study combines a fast produced plant-based HA antigen (HAC1), an NP-based drug delivery system, and an adequate test system to reflect local effects of an influenza vaccine. Firstly, we determined that the vaccine (HAC1-NP) was not toxic in human PCLS. Secondly we analyzed the cell specific responses to the vaccine in lung tissue sections on the cytokine level. Our data established a safe, non-toxic concentration range of the plant-based produced HAC1 formulated with NP. Within these concentrations the HAC1-NP vaccine induces an innate immune stimulation and re-activates a specific T cell response in the human lung tissue.

## Materials and Methods

### Ethics Statement

PCLS experiments using human tissue were approved by the ethics committee of the Hannover Medical School and are in accordance with *The Code of Ethics of the World Medical Association*. All patients or their next of kin, caretakers, or guardians gave written informed consent for the PCLS experiments.

### Culture Media and Reagents

All media and reagents were obtained from Sigma Aldrich (Munich, Germany) if not stated otherwise. Dulbecco’s Modified Eagle’s Medium Nutrient Mixture F-12 Ham (DMEM, pH 7.2–7.4) with L-glutamine and 15 mM HEPES, but without phenol red and fetal bovine serum, was complemented with 7.5% (w/v) sodium bicarbonate and 100 U/mL penicillin and with 100 µg/mL streptomycin. PBS (0.1 M sodium phosphate and 0.15 M NaCl, without Ca^2+^ and Mg^2+^, pH 7.4) was purchased from Lonza (Verviers, Belgium). Lipopolysaccharide (LPS) of *E. coli*, serotype 0111:B4 was supplied in the lyophilized form and dissolved in PBS (Lonza). The Wst-1 assay kit was obtained from Roche (Mannheim, Germany) and the BCA total protein kit was obtained from Thermo Scientific (Rockford, IL, USA).

### Antigen and Nanoparticles

The recombinant HA antigen (HAC1) of the A/California/4/09 (H1N1) influenza strain was expressed in tobacco plants as described previously in detail [29], [30]. The *Agrobacterium tumefaciens* was transfected with a construct of a helper plasmid and a plant virus expression vector, cloned with parts of the HA sequence (AA 18–530). After cultivation, the transformed agrobacteria were infiltrated into greenhouse-grown 6-week-old *Nicotiana benthamiana* plants. The plant tissue was harvested and homogenized after 7 days. Subsequently, HAC1 was purified by immobilized metal affinity and anion exchange chromatography to a purity of >90%.

For the generation of silica-NP, aqueous nanosilica formulations were prepared from SiO_2_ nanopowder (HDK 200, Wacker Chemie, Germany) in DMEM using an ultrasonic sonotrode as dispersion aid. The average particle size distribution for 0.1% SiO_2_ nanopowder (hydrodynamic count mode diameter 100 nm) was stable for at least 4 hours. The antigen and the silica-NP were premixed on an overhead shaker for 10 min prior to use. To investigate the binding of the silica-NP and the antigen (ratio 1∶100) formulations were prepared and centrifuged at 3000 rpm (∼755×g) for 4 min. The supernatant was removed from the easily visible silica-pellet. The Pellet and supernatant samples were filled up with PBS to 0.5 ml and frozen until BCA analysis of the protein content in the different phases. 83–99% of the used protein were recovered in the silica-pellet phase indicating a high antigen-binding capacity of the silica-NP at the used ratio.

### Human Donors

The lung lobes were obtained from patients who underwent surgical resection at the Hannover Medical School or the KRH Klinikum Oststadt-Heidehaus. Only tumor-free lung tissue was used. The specimens were processed immediately after resection. The average age of patients was 59±8 years (one exception: 8 years old) (Table S1). The number of patients has been indicated in each figure.

### Preparation of PCLS

Human PCLS were prepared as previously described in detail [27], [31]. Briefly, human lungs were cannulated with a flexible catheter and selected segments were gently inflated with warm (37°C) medium (DMEM containing 1.5% liquid, low-gelling agarose (Sigma Aldrich, Munich, Germany)). After the agarose polymerized on ice, tissue segments were cut with a rotating sharpened metal tube (∅ 8 mm). The sections were sliced with a Krumdieck tissue slicer (Alabama Research and Development, Munford, AL, USA) into approx. 250 µm thin slices in Earl`s Balanced Salt Solution (EBSS, Sigma Aldrich). PCLS were washed 3 to 4 times and cultivated in DMEM under normal cell culture conditions (37°C, 5% CO_2_, and 100% air humidity). For different treatments, PCLS were incubated with DMEM containing different concentrations of silica-NP, HAC1 or a combination of both (HAC1-NP) under submerse cell culture conditions. Additionally, untreated PCLS were used as a reference. PCLS treated with 1% Triton X-100 served as a death control, whereas LPS (100 ng/mL) treated PCLS served as a positive control. Supernatants were collected after 24 hours of incubation, supplemented with 0.2% protease inhibitor cocktail P1860 (Sigma Aldrich), and stored at −80°C until further analyses.

### Wst-1 Assay

The metabolic activity was assessed by the Wst-1 assay according to the manufacturer’s protocol. After 24 hours of treatment, the supernatant was removed and supplemented with 0.2% protease inhibitor cocktail before storage. PCLS were incubated for 1 hour at 37°C with 0.125 mL of Wst-1 solution per slice (1∶10 in culture medium). The intensity of the soluble formazan was measured at a wavelength of 420–480 nm with a reference wavelength of 690 nm.

### Calcein AM/Ethidium Homodimer-1 Staining (“LIVE/DEAD® Staining”)

Toxic effects of treatments were determined by viability staining of the slices using the LIVE/DEAD® Viability/Cytotoxicity kit (Life technologies, Darmstadt, Germany) according to the manufacturer’s protocol as previously described [27]. The enzymatic conversion of calcein AM to intensely green fluorescent calcein is associated with the esterase activity in living cells. Dead cells were distinguished by EthD-1 binding to DNA, producing an intracellular orange/red fluorescence which is a marker of the loss of plasma membrane integrity.

After a 24 hour treatment, PCLS were incubated with 4 µM calcein AM and 4 µM ethidium homodimer-1 (EthD-1) for 45 min at room temperature (RT) on an orbital shaker (150 rpm). The slices were washed in DMEM and analyzed by a confocal laser scanning microscope Meta 510 (Zeiss, Jena, Germany). From each treated slice, random triplicates from 30 µm thick 3D stacks were recorded (10×objective, excitation wavelengths 488 nm and 543 nm, emission filters LP 560 nm and BP 505–550 nm) and quantitatively analyzed using the IMARIS 7.4.0 software (Bitplane Scientific Software, Zurich, Switzerland).

### Quantitative Image Analysis

The computer-based quantitative analysis of LIVE/DEAD® staining was performed using the IMARIS 7.4.0 software as described previously [27]. Briefly, the total volume of calcein fluorescencent structures (green channel) was calculated in the confocal datasets, with a dimension of 900×900×30 µm, equating the volume of the cytoplasm of viable cells. In the second step, the nuclei (approx. 5 µm in diameter) of the dead cells stained with EthD-1 (red channel) were counted via the semi quantitative “surface rendering” software tool. The subsequent adjustments (exclusion of intra-alveolar macrophages, threshold, etc.) for each channel were performed on the reference tissue and adopted unchanged for all datasets of all treatment groups.

For the quantitative analysis, the ratio of the counted dead cell nuclei and the volume of the cytoplasm of the living cells were calculated (dead cell nuclei/10^6^ µm^3^ cytoplasm volume).

### Measurement of Cytokine Levels by Meso Scale Discovery Technology

For the measurement of Tumor necrosis factor-alpha (TNF-α), Interleukin (IL)-8, IL-1 beta, Interferon-gamma (IFN-γ), IL-2, IL-4, IL-5, IL-13, IL-12 heterodimer (IL-12(p70)) and IL-10 in the supernatants of the differently treated PCLS, the human T_h_1/T_h_2 (10-plex) tissue culture Kit from Meso Scale Discovery (MSD) Assays (Gaithersburg, MD, USA) was used. The assay was performed according to the manufacturer’s specifications using MSD plates and the MSD Sector Imager 2400. The calculation of the cytokine concentrations was based on a 4-fold serial diluted standard. Data analyses were conducted using the discovery workbench software.

Total protein concentrations in human PCLS were determined with the BCA Protein Assay kit according to the manufacturer’s instructions. The level of each cytokine was related to the total protein content (pg cytokine/mg total protein) to quantify and exclude any variations of the slice thickness.

### Fluorescence Staining of CD3^+^ T cells in Human PCLS

PCLS were fixed in 2% PFA for 24 h and stored in sodium azide at 4°C. Before staining PCLS were washed, permeabilized with 0.3% Triton X-100 in PBS for 1 h and immunostained as described before for whole mount staining [32]. Briefly, PCLS were pre-blocked with PBS-Blocker (PBS suppl. with 0.5% bovine serum albumin and 4% donkey serum) for 1 h. The primary monoclonal CD3 antibody (# 344801, Biolegend, San Diego, USA) was diluted 1∶50 in PBS-Blocker to a final concentration of 10 µg/ml. Isotype control was Mouse IgG1, κ (# 400101, Biolegend, San Diego, USA). PCLS were incubated with the first antibody overnight at 4°C. After five 20-minute washes in PBS +0.05% Tween20 PCLS were blocked with PBS-Blocker for 1 h, before they were incubated with the secondary donkey anti-mouse antibody labeled with Cy3 (1∶400, Jackson ImmunoResearch, West Grove, PA) overnight at 4°C. After washing, the nuclei were stained for 10 minutes with 2 µM To-Pro®-3 iodide (1∶500, # T3605, Molecular Probes, Life technologies, Darmstadt, Germany). Before the samples were mounted in Prolong Gold mounting medium (Molecular Probes, Eugene, OR) and analyzed with Zeiss confocal microscope (LSM510, 40× water-objective) the PCLS were washed three-times for 10 minutes in PBS-Tween20 and rinsed with ultrapure distilled H_2_O for reduction of background signals.

### Statistical Analysis

Statistical analysis was performed using GraphPad 4.03 for Windows (GraphPad, San Diego, CA). The data were expressed as mean ± standard error of the mean (SEM). The statistical analysis was performed by Friedman test and Dunn’s Multiple Comparison Post-hoc tests. Differences between treatment groups and controls were considered statistically significant at a level of p<0.05. The number of patients is indicated in the figure legends.

## Results

### Plant-produced Influenza Vaccine HAC1-NP is not Toxic to Human Lung Tissue at Low Concentrations

**Figure 1:**
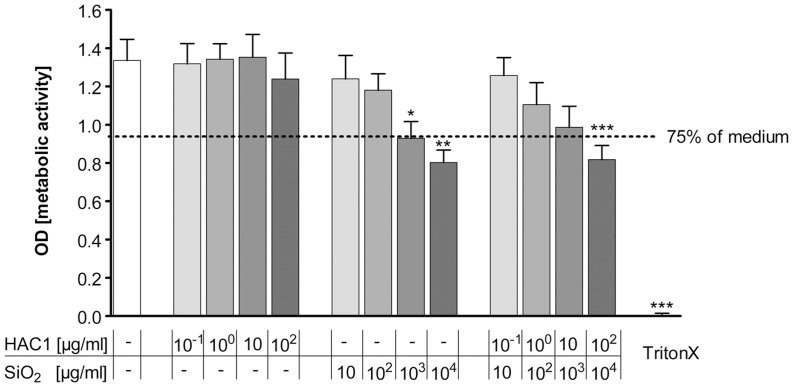
Effects of the recombinant hemagglutinin protein HAC1 and SiO_2_ on metabolic activity of human PCLS. Increasing concentrations of the plant-derived recombinant hemagglutinin protein HAC1 from the H1N1 virus had no significant influence on the metabolic activity of the human lung tissue. In contrast to HAC1, the SiO_2_ nanoparticles had in higher concentrations (≥10^3^ µg/ml) a significant effect on the metabolic activity in human PCLS compared to the tissue control after 24 hours. Similarly the combined treatment (HAC1+ SiO_2_ nanoparticles) also caused in the highest concentrations (10^2^ µg/ml HAC1 bound onto 10^4^ µg/ml SiO_2_) a significant decrease in the metabolic activity of the cells. Data are presented as mean±SEM, *p<0.05, **p<0.01 compared to untreated tissue control, Friedman test and Dunn’s Multiple Comparison Post-hoc test (n = 13). Doted line marks 75% of the tissue control. PCLS = precision cut lung slices, HAC1 =  plant-derived recombinant hemagglutinin protein, SiO_2_ = silica nanoparticles.

In order to determine safe, non-toxic concentrations of HAC1-NP, the metabolic activity of the human lung tissue was measured by the Wst-1 assay after 24 hours exposure to HAC1, silica-NP and the formulated vaccine HAC1-NP. Increasing concentrations of HAC1 revealed no effect on the metabolic activity in the human lung tissue compared to the non-exposed tissue control (Fig. 1). In contrast, silica-NP induced a significant decrease in the metabolic activity of the lung tissue at concentrations of ≥10^3^ µg/ml (Fig. 1). In line with the results of the single components, the formulated vaccine HAC1-NP showed no significant decrease in the metabolic activity at lower concentrations (<10^4^ µg/ml NP formulated with 10^2^ µg/ml HAC1; Fig. 1).

**Figure 2:**
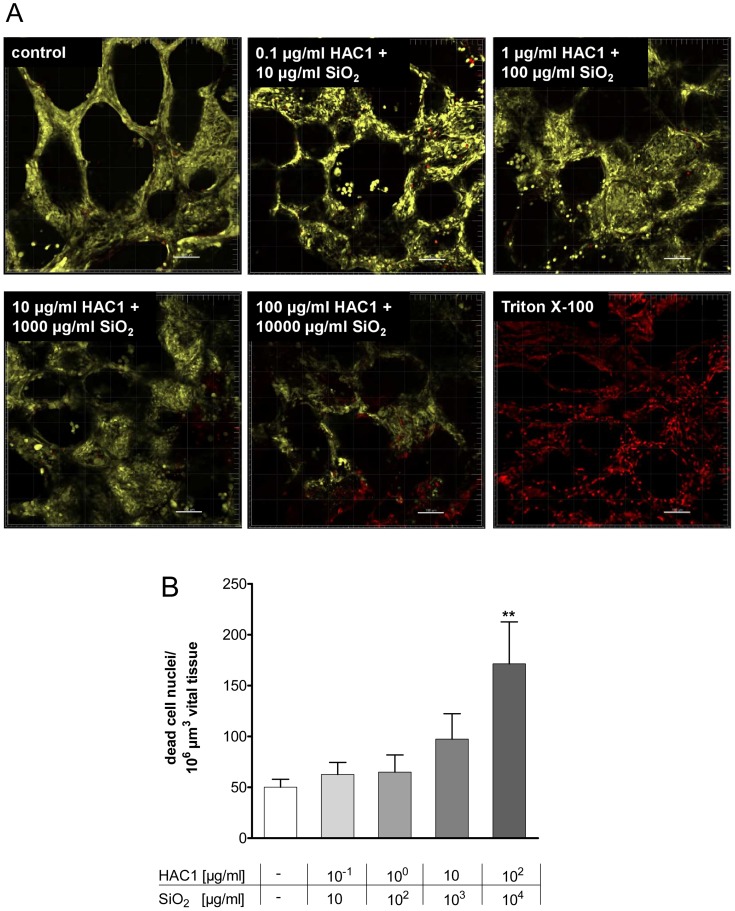
Three dimensional detection and semi-quantitative image analysis of viability staining after 24 h of incubation with HAC1 bound onto SiO_2_ nanoparticles in human PCLS. Human lung slices were treated without (control) or with increasing concentrations of the plant-derived recombinant hemagglutinin protein HAC1 bound onto increasing concentrations of SiO_2_ nanoparticles (ratio HAC1:SiO_2_ = 1∶100) or with triton X as a negative control (A). The images were analyzed with the IMARIS 5.5.3. Software and semi-quantitatively evaluated (B). Viability of PCLS is expressed as quantity of spots (>4 µm diameter) in 10^6^ µm^3^ yellow tissue volume. Data are presented as mean±SEM, **p<0.01 compared to untreated tissue control, Friedman test and Dunn’s Multiple Comparison Post-hoc test (n = 11). HAC1 = plant-derived recombinant hemagglutinin protein, SiO_2_ = silica nanoparticles.

The results of the Wst-1 assay were independently confirmed by LIVE/DEAD® staining using confocal microscopy. Here, human PCLS treated with increasing concentrations of HAC1-NP revealed no loss of viability up to 10^4^ µg/ml NP and 10^2^ µg/ml HAC1 after the 24 hours exposure (Fig. 2A). Quantitative image analysis showed no toxic effects of the lung tissue by concentrations ≤10^3^ µg/ml NP and 10 µg/ml HAC1, but viability decreased significantly at concentrations >10^3^ µg/ml NP and 10 µg/ml HAC1 (Fig. 2B).

### Treatment with HAC1-NP Minimally Induces the Pro-inflammatory Cytokine TNF-α in PCLS

**Figure 3:**
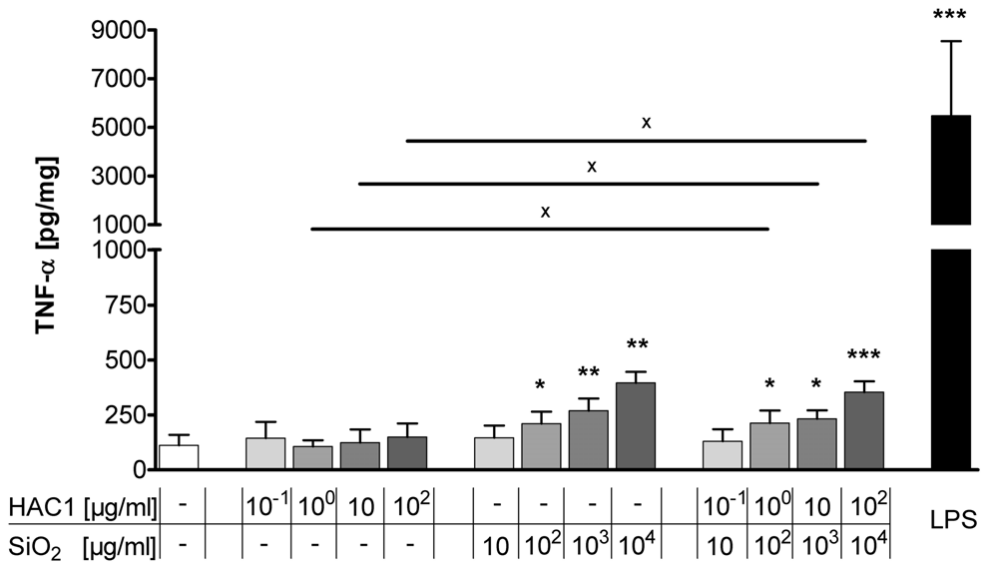
Extracellular release of the pro-inflammatory cytokine TNF-α in human PCLS after 24 h of treatment with HAC1, SiO_2_ or HAC1-SiO_2_. Human PCLS were treated without (control) or with increasing concentrations of either the plant-derived recombinant hemagglutinin protein HAC1 or the SiO_2_ nanoparticles or the protein bound onto SiO_2_ nanoparticles (ratio HAC1:SiO2 = 1∶100) or with LPS as an inflammatory control. The cytokine levels of TNF-α in PCLS culture supernatants were determined by Multiplex MSD technology. Data are presented as mean±SEM, *p<0.05, **p<0.01 & ***p<0.001 compared to untreated tissue control, ^X^p<0.05 compared to corresponding concentration of combined treatment (HAC1 vs. HAC1-SiO_2_ or SiO_2_ vs. HAC1-SiO_2_), Friedman test and Dunn’s Multiple Comparison Post-hoc test (n = 13). HAC1 =  plant-derived recombinant hemagglutinin protein, SiO_2_ = silica nanoparticles.

The acute inflammatory immune response in the human lung tissue to HAC1, silica-NP and HAC1-NP was analyzed after 24 hours of exposure. TNF-α was chosen as a pro-inflammatory marker and was measured in the supernatants of PCLS (Fig. 3). The plant-produced protein HAC1 did not affect the secretion of TNF-α in the lung tissue. In contrast, treatments of the lung tissue with silica-NP or with HAC1-NP significantly increased TNF-α secretion at concentrations >10^2^ µg/ml silica-NP in a dose-dependent manner compared to reference or HAC1 protein treated tissue. Treatment with LPS induced a mean 75-fold increase in the TNF-α secretion compared to the untreated tissue. Furthermore the LPS treatment compared to the HAC1-NP and silica-NP treatment induced an average increase of TNF-α by 23-fold and 18-fold, respectively.

### Treatment with HAC1-NP Induces the Pro-inflammatory Cytokine IL-1β in Human PCLS

**Figure 4:**
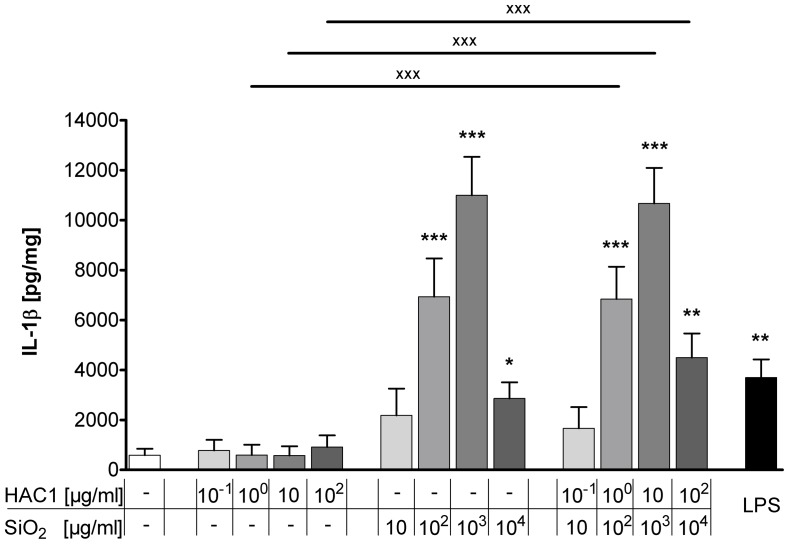
Extracellular IL-1β release in human PCLS after 24 h treatment with HAC1, SiO_2_ or HAC1-SiO_2_. Human PCLS were treated without (control) or with increasing concentrations of either the plant-derived recombinant hemagglutinin protein HAC1 (A) or the SiO_2_ nanoparticles (B) or a combination of both (ratio HAC1:SiO2 = 1∶100; C) or with LPS as an inflammatory control. The cytokine levels of IL-1β in PCLS culture supernatants were determined by Multiplex MSD technology. Data are presented as mean±SEM, *p<0.05, **p<0.01compared to untreated tissue control,^ XXX^p<0.001 compared to corresponding concentration of combined treatment (HAC1 vs. HAC1-SiO_2_ or SiO_2_ vs. HAC1-SiO_2_) Friedman test and Dunn’s Multiple Comparison Post-hoc test (n = 13). HAC1 =  plant-derived recombinant hemagglutinin protein, SiO_2_ = silica nanoparticles.

In order to investigate the silica-NP-specific induction of an innate immune response and the associated adjuvant property, the release of another pro-inflammatory cytokine, IL-1β, was measured in the tissue culture supernatant (Fig. 4). After 24 hours of treatment, HAC1 did not affect the release of IL-1β at any concentration compared to the IL-1β baseline levels. In contrast, silica-NP dose-dependently increased IL-1β secretion. The baseline IL-1β level of 585±261 pg/mg was elevated to 10996±1545 pg/mg when the lung tissue was treated with 10^3^ µg/ml NP. Likewise, the formulated vaccine HAC1-NP increased IL-1β release in PCLS. The treatment with a concentration of 10^3^ µg/ml NP and 10 µg/ml HAC1 elevated the baseline IL-1β content to 10674±1423 pg/mg. The comparison between correlating concentrations of HAC1 and the formulated vaccine HAC1-NP showed a significant increase of IL-1β induced by HAC1-NP at concentrations ≥10 µg/ml HAC1 formulated with 10^2^ µg/ml silica-NP.

### Treatment with HAC1-NP Induces an Antigen-specific T cell Response in PCLS

The T cell-specific cytokines IL-2 and IFN-γ have been measured to elucidate whether the new vaccine HAC1-NP is able to induce a specific T cell response.

**Figure 5:**
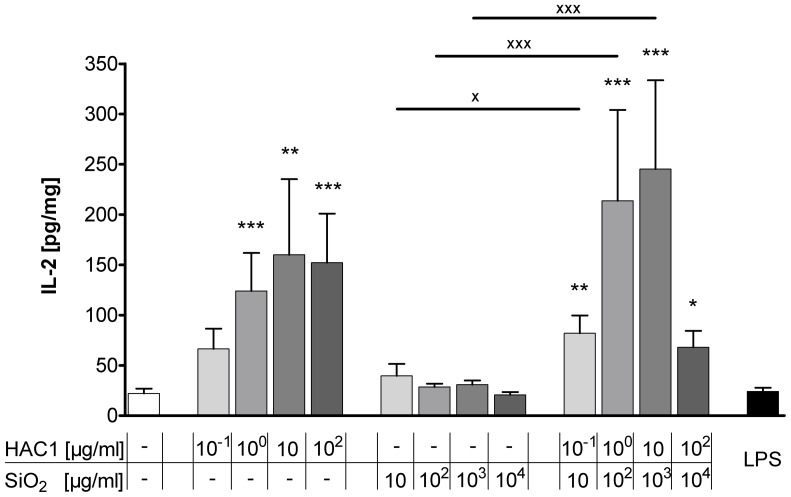
Release of extracellular IL-2 in human PCLS after 24 h treatment with the test substances. Human PCLS were treated without (control) or with increasing concentrations of either the plant-derived recombinant hemagglutinin protein HAC1 or the SiO_2_ nanoparticles or a combination of both (ratio HAC1:SiO2 = 1∶100) or with LPS. The cytokine levels of IL-2 in PCLS culture supernatants were determined by Multiplex MSD technology. Data are presented as mean±SEM, *p<0.05, **p<0.01 & ***p<0.001 compared to untreated tissue control, ^X^p<0.05 & ^XXX^p<0.001 compared to corresponding concentration of combined treatment (HAC1 vs. HAC1-SiO_2_ or SiO_2_ vs. HAC1-SiO_2_), Friedman test and Dunn’s Multiple Comparison Post-hoc test (n = 13). HAC1 =  plant-derived recombinant hemagglutinin protein, SiO_2_ = silica nanoparticles.

**Figure 6:**
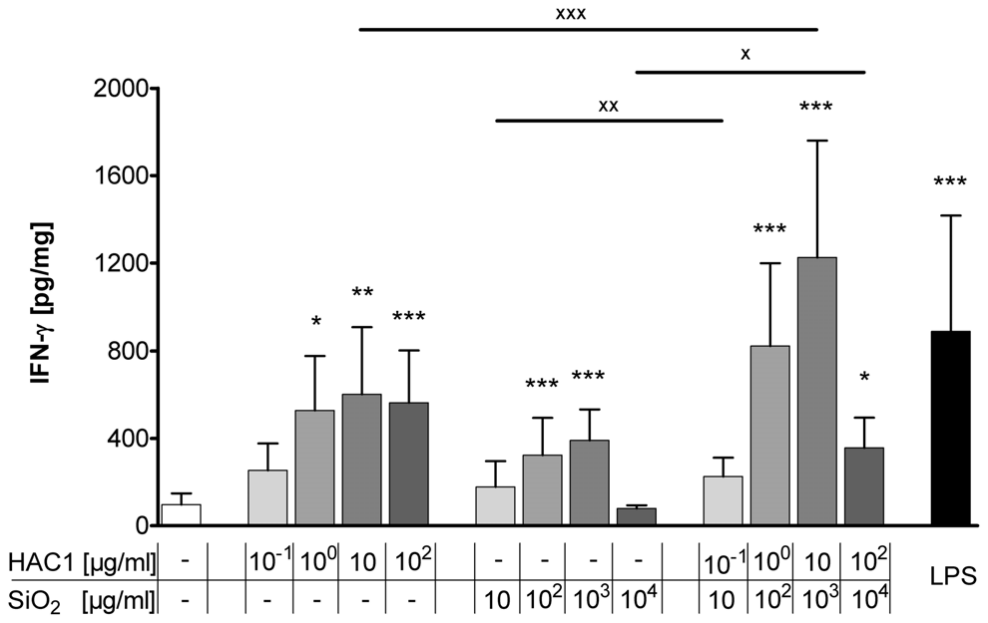
Release of extracellular Interferon-gamma in human PCLS after 24 h treatment with the test substances. Human PCLS were treated without (control) or with increasing concentrations of either the plant-derived recombinant hemagglutinin protein HAC1 or the SiO_2_ nanoparticles or a combination of both (ratio HAC1:SiO2 = 1∶100) or with LPS. The cytokine levels of Interferon-gamma (IFN-γ) in PCLS culture supernatants were determined by Multiplex MSD technology. Data are presented as mean±SEM, *p<0.05, **p<0.01 & ***p<0.001 compared to untreated tissue control, ^X^p<0.05, ^XX^p<0.01 & ^XXX^p<0.001 compared to corresponding concentration of combined treatment (HAC1 vs. HAC1-SiO_2_ or SiO_2_ vs. HAC1-SiO_2_), Friedman test and Dunn’s Multiple Comparison Post-hoc test (n = 13). HAC1 =  plant-derived recombinant hemagglutinin protein, SiO_2_ = silica nanoparticles.

After the 24 hour incubation with the test substances, alone HAC1 and HAC1-NP induced a significant dose-dependent increase in extracellular IL-2, the T cell proliferation cytokine (Fig. 5). In contrast, silica-NP only had no effect on the release of IL-2. Furthermore, the anti-viral T helper cells type 1 (T_h_1) cytokine IFN-γ was dose-dependently increased by HAC1 and HAC1-NP (Fig. 6). A concentration of 10 µg/ml HAC1 significantly increased the level of extracellular IFN-γ compared with the reference control level of 97±51 pg/mg to 602±307 pg/mg (p<0.01). The highest non-toxic silica-NP concentration (10^3^ µg/ml) also significantly increased the IFN-γ release (97±51 pg/mg to 391±141 pg/mg; p<0.001). Comparable to HAC1, the formulated vaccine HAC1-NP also dose-dependently increased the IFN-γ release. A concentration of 10 µg/ml HAC1 and 10^3^ µg/ml NP significantly increased the baseline IFN-γ content from 97±51 pg/mg to 1226±535 pg/mg (p<0.001). Furthermore the formulated vaccine HAC1-NP significantly increased the IFN-γ secretion at 10 µg/ml formulated with 10^3^ µg/ml NP compared to the treatment with the HAC1 protein alone (1226±535 pg/mg vs. 602±307 pg/mg; p<0.001). In contrast, production of T_h_2-type cytokines IL-4, IL-5, and IL-13 was not secreted at biologically significant levels (Table S2).

## Discussion

This study aimed to characterize a new respiratory influenza vaccine formulated with a NP-based drug delivery system. A human relevant *ex vivo* model was used to test its local toxcicity, as well as its potential to recall an immune response, and showed a re-activation of a specific T cell response induced by the protein accompanied by a silica-NP-dependent adjuvant effect.

According to the WHO recommendations, vaccination is still the gold standard to prevent influenza infections [1]. However, as demonstrated during the H1N1 pandemics in 2009, the existing influenza vaccines have limitations [33], [34]. Respiratory vaccination offers a reasonable alternative for the common systemic vaccination to enhance the immune response, targeting the main path of the influenza infection and settlement. Madhun and colleagues provided evidence that vaccination via the nasal route increased not only virus-specific serum IgG levels, but induced local IgA production [18].

Whereas these findings focused on vaccination only via the upper respiratory tract, in the present study we were interested in a local vaccine administration targeting the whole respiratory tract. Since influenza usually infects the whole respiratory tract and some virus strains even tend to bind deeply in the lower respiratory tract in humans [35], this approach is expected to result in stronger and broader protection against infection. As the lung has a larger surface compared to the nasal tract, this results in a better bioavailability of vaccines to cells, but also in a presumably higher sensitivity to toxic side effects. In our well-established *ex vivo* model of human lung tissue the Wst-1 assay and the Live/Dead® staining analysis revealed a non-toxic window of HAC1-NP with up to 10 µg/ml HAC1 formulated with 10^3^ µg/ml SiO_2_. An analysis of the metabolic activity of all vaccine components showed that the toxic effects were exerted by silica-NP only. However, these toxic concentrations (10^3^–10^4^ µg/ml SiO_2_) are well above a presumable therapeutic dose.

Additionally the key marker of pro-inflammatory effects, TNF-α, was not induced by the protein alone, but by the silica–NP in a dose-dependent manner. Moreover this inflammatory effect was also reflected by the dose-dependent TNF-α increase induced by the formulated vaccine HAC1-NP. Interestingly, the mean silica-NP-induced TNF-α secretion was 13-times lower than the secretion induced by the major pro-inflammatory stimulus LPS. In contrast, the release of the pro-inflammatory mediator IL-1β induced by the silica-NP considerably exceeded the LPS-induced release. These results suggest that the silica-NP and LPS may have triggered different inflammatory pathways or activated different cell types. Previously, it has been reported that silica possesses adjuvant properties in guinea pigs and mice [36], [37]. Moreover, silica-NP were shown to activate the NLRP3 inflammasome, resulting in the release of IL-1β [38], [39]. In line with this, we observed a silica-NP-specific and dose-dependent induction of IL-1β which was not found for HAC1. Secretion of IL-1β significantly decreased at the highest concentration of SiO_2_ (10^4^ µg/ml)_,_ which might be explained by the decreased lung tissue vitality and metabolic activity. The fact that activation of TNF-α and IL-1β in our study was only induced by the silica-NP (alone or formulated as the vaccine) points to silica-specific adjuvant properties also in humans. During PCLS preparation, some cellular damage occurs and mediators are released. However to minimize a triggering of pro-inflammatory responses by intracellular contents, the PCLS were intensively washed before the treatments to remove cell debris and apoptotic and inflammatory mediators. After a couple of hours the intracellular content in the supernatant returned to baseline levels. Especially the baseline levels of the major pro-inflammatory cytokines TNF-α and IL-1β for the untreated medium control did not exceed 112±48 pg/mg and 585±261 pg/mg respectively. It is important to mention that some cytokine baseline levels, for example for IL-8, are very high (data not shown). These high levels might be explained by the pathology status of the patients.

In any case, activation of the tissue can not be excluded *per se*. Yet, all experiments were compared to their own tissue-specific internal medium control to discriminate between treatment-specific and preparation-specific effects. Furthermore it has been demonstrated previously that the PCLS response to inflammatory stimuli reflects the *in vivo* situation exceedingly well [27]. Switalla and colleagues showed that the LPS-induced cytokine profile of human PCLS highly correlates with that from LPS-provoked human BAL fluid [27]. These results demonstrate the reliability of the PCLS method for measuring non-artificial effects induced for example by the cutting process itself.

Besides studying toxic and pro-inflammatory effects of the new vaccine formulation, we explored the vaccines potential to trigger or re-activate an immune response. An effective vaccination requires generation of both strong humoral and cellular immune responses [40], [41]. The humoral immune response is crucial for the protection against influenza infections via antibody-mediated neutralization of the virus. The cellular immunity relies mainly on the induction of CD4^+^ T_h_ cells and CD8^+^ cytotoxic T cells (CTL) and is essential for viral clearance [42]. The differentiation of naÏve CD4^+^ T cells into T_h_1 cells or T_h_2 cells depends on the local cytokine milieu [43]. T_h_1 cells, characterized by their key cytokine IFN-γ, are pivotal for the stimulation of CTL responses and the induction of memory CD8^+^ T cells [44]. Activated CTLs eliminate influenza virus-infected cells [45]. It has also been shown that memory CD4^+^ T cells account decisively for a faster control of influenza infection on repeated exposure to the virus [46], [47]. Purwar and colleagues demonstrated that resident T cells are abundant in the human lung (>10^10^) [48]. In line with this, our fluorescence staining confirmed the presence of CD3^+^ T cells in human PCLS (Figure S1). Further Purwar et al. reported that there were abundant numbers of resident memory T cells (T_RM_) in the lung parenchyma, characterized by the expression of specific markers (CD45RO^+^, CD3^+^, CD45RA^-^, CD4^+^ or CD8^+^, CD25, CD69, HLA-DR, TCRαβ^+^, α4β7^-^, CCR5, CXCR3, CXCR4, β1-integrin VLA-1 and PSGL-1). The majority of CD4^+^ T_RM_ were found to be multifunctional T_h_1 type cells secreting IFN-γ and IL-2 upon restimulation [48]. Moreover these lung T_RM_ proliferate in response to the influenza virus [48]. The authors suggested that these T_RM_ cells are available for a recall response at a repeated mucosal encounter with a known antigen. Similarly, our study has demonstrated a dose-dependent induction of the T cell activation and proliferation marker IL-2 in PCLS after exposure to the vaccine. The effect was dependent on the influenza antigen and was not observed with the silica-NP. This antigen-specific IL-2 induction indicates a previous encounter of the mucosal lung tissue with either the H1N1 virus or to another cross-reactive influenza strain, which led to the development of antigen-specific lung T_RM_ in the PCLS. The formulated vaccine would have re-activated these T_RM_ cells and induced a secondary immune response. This is further supported by the observation that IFN-γ was also significantly induced by the formulated vaccine. Interestingly, the levels of IFN-γ induced by the formulated vaccine (10 µg/ml HAC1 and 10^3^ µg/ml SiO_2_) exceeded those induced by the single components (10 µg/ml HAC1 or 10^3^ µg/ml SiO_2_). This boosted memory T cell re-activation supports the adjuvantic qualities of the sub-toxic silica-NP concentrations in human PCLS. Additionally, we have observed an antigen-independent release of IFN-γ in response to the silica-NP. This may have occurred because of triggering a different molecular pathway compared with that involved in the antigen-dependent IFN-γ release by CD4^+^ T_RM_. As mentioned before, silica activates the NLRP3 inflammasome which releases IL-1β and IL-18. IL-18, originally known as IFN-γ-inducing factor, provides an important link between the innate and adaptive immune responses and might explain the silica-NP-induced IFN-γ secretion detected in our PCLS system.

The HAC1-induced cytokine profile in our *ex vivo* study matches perfectly with the results described by Jul-Larsen and colleagues [30]. They also described significant HAC1 induction of IL-2 and IFN-γ secretion in multifunctional CD4^+^ T cells isolated from the blood of volunteers vaccinated with the 2009 pandemic H1N1 vaccine. Even higher cytokine secretion was achieved when PBMCs were treated with the vaccine antigen. Based on these results, the authors suggested that a HAC1 vaccine may require the presence of additional immunostimulatory agents to induce a robust T cell response [30]. In conclusion, our results demonstrate that PCLS as an organotypic *ex vivo* model of the human respiratory tract can be used to study vaccine-induced re-activation of lung memory T cells and the innate immune system. Furthermore this study showed the adjuvant effect of the NP-based drug delivery system, and demonstrated a boosted recall immune response to the formulated influenza vaccine within non-toxic concentrations on human lung tissue.

## Supporting Information

Figure S1 **Fluorescence staining of CD3^+^ T cells in human lung tissue slices.** Human PCLS were stained with a CD3 specific antibody (A; Red) or an Isotype control antibody (B) and the nuclei specific marker ToPro3 (Blue) to detect T cells within the tissue. There were CD3 positive cells detectable in the human PCLS (Arrows).
(DOCX)

Table S1 **Tissue related patient information used for the human PCLS.** Information about the tissue used for the PCLS experiments regarding age, gender, lung lobe removed and reason for lobectomy. Yrs = Years;
(DOCX)

Table S2 **Release of extracellular cytokines in human PCLS after 24 h treatment with the test substances.** Human PCLS were treated with medium or with increasing concentrations of either the plant-derived recombinant hemagglutinin protein HAC1 or the SiO_2_ nanoparticles or a combination of both HAC1-NP (ratio HAC1:SiO2 = 1∶100) or with LPS. The cytokine levels in PCLS culture supernatants of Interleukine-2 (IL-2), Interferon-gamma (IFN-γ), Interleukine 1 beta (IL-1β), Tumor necrosis factor alpha (TNF-α), Interleukine 4 (IL-4), Interleukine 5 (IL-5), Interleukine 13 (IL-13), Interleukine 12p70 (IL-12p70) and Interleukine 10 (IL-10) were determined by Multiplex MSD technology. The total protein content in the supernatant was determined by BCA assay. To quantify variations of the slice thickness and their associated variations the cytokine content was related to the total protein content (pg cytokine/mg total protein). Data are presented as mean±SEM, n = 13.
(DOCX)
