# Supplementary figures and images for: Functional Testing of an Inhalable Nanoparticle Based Influenza Vaccine Using a Human Precision Cut Lung Slice Technique
Source: PLoS One. 2013 Aug 13;8(8):e71728. doi: 10.1371/journal.pone.0071728 (PMC3742667; doi:10.1371/journal.pone.0071728)

**
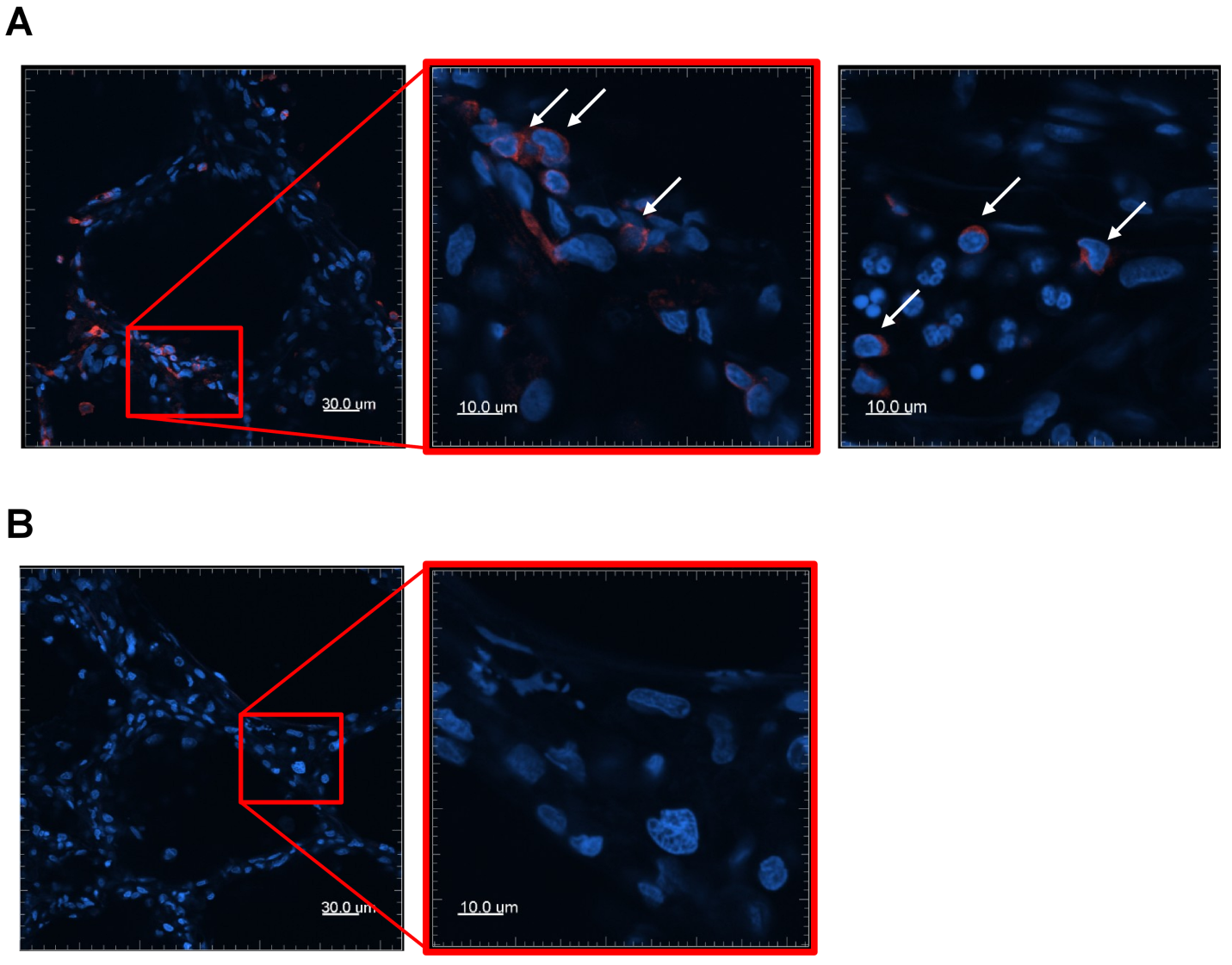
**

Supplement: Figure S1 — Fluorescence staining of CD3+ T cells in human lung tissue slices. Human PCLS were stained with a CD3 specific antibody (A; Red) or an Isotype control antibody (B) and the nuclei specific marker ToPro3 (Blue) to detect T cells within the tissue. There were CD3 positive cells detectable in the human PCLS (Arrows). (DOCX) [file pone.0071728.s001.docx]
