# Supplementary material for: Functional Testing of an Inhalable Nanoparticle Based Influenza Vaccine Using a Human Precision Cut Lung Slice Technique
Source: PLoS One. 2013 Aug 13;8(8):e71728. doi: 10.1371/journal.pone.0071728 (PMC3742667; doi:10.1371/journal.pone.0071728)
Supplement: Table S1 — Tissue related patient information used for the human PCLS. Information about the tissue used for the PCLS experiments regarding age, gender, lung lobe removed and reason for lobectomy. Yrs = Years; (DOCX) [file pone.0071728.s002.docx]

| **Nummer** | **Patient**  **(Male/ Female; age)** | **Lung lobe/ disease** |
| --- | --- | --- |
| 1 | Male / 67 Yrs | Right upper lobe/  Squamous cell carcinoma |
| 2 | Male / 62 Yrs | Left lung lobe/ Emphysema |
| 3 | Female / 44 Yrs | Left lung lobe/ Emphysema |
| 4 | Male / 60 Yrs | Right upper lobe/ Fibrosis |
| 5 | Female / 71 Yrs | Right upper lobe/ Squamous cell carcinoma |
| 6 | Female / 53 Yrs | Right lobe/ Fibrosis |
| 7 | Male / 62 Yrs | Right lung lobe/ Emphysema |
| 8 | Female / 54 Yrs | Left lung lobe/ Fibrosis |
| 9 | Male/ 8 Yrs | Right lung lobe/ Idiopath. pulmonary hypertonia |
| 10 | Male/ 63 Yrs | Right lower lobe/ Fibrosis |
| 11 | Female / 52 Yrs | Right lower lobe/ Tumor |
| 12 | Female / 53 Yrs | Right lower lobe/ Adenocarcinoma |
| 13 | Male / 64 Yrs | Right upper lobe/ Tumor |
