# Supplementary material for: Functional Testing of an Inhalable Nanoparticle Based Influenza Vaccine Using a Human Precision Cut Lung Slice Technique
Source: PLoS One. 2013 Aug 13;8(8):e71728. doi: 10.1371/journal.pone.0071728 (PMC3742667; doi:10.1371/journal.pone.0071728)
Supplement: Table S2 — Release of extracellular cytokines in human PCLS after 24 h treatment with the test substances. Human PCLS were treated with medium or with increasing concentrations of either the plant-derived recombinant hemagglutinin protein HAC1 or the SiO2 nanoparticles or a combination of both HAC1-NP (ratio HAC1:SiO2 = 1∶100) or with LPS. The cytokine levels in PCLS culture supernatants of Interleukine-2 (IL-2), Interferon-gamma (IFN-γ), Interleukine 1 beta (IL-1β), Tumor necrosis factor alpha (TNF-α), Interleukine 4 (IL-4), Interleukine 5 (IL-5), Interleukine 13 (IL-13), Interleukine 12p70 (IL-12p70) and Interleukine 10 (IL-10) were determined by Multiplex MSD technology. The total protein content in the supernatant was determined by BCA assay. To quantify variations of the slice thickness and their associated variations the cytokine content was related to the total protein content (pg cytokine/mg total protein). Data are presented as mean±SEM, n = 13. (DOCX) [file pone.0071728.s003.docx]

| **Treatment** | **[µg/ml]** | **IL-2 [pg/mg]** | **INF-γ **  **[pg/mg]** | **IL-1β **  **[pg/mg]** | **TNF-α **  **[pg/mg]** | **IL-4 [pg/mg]** | **IL-5 [pg/mg]** | **IL-13 [pg/mg]** | **IL-12p70 [pg/mg]** | **IL-10 [pg/mg]** |
| --- | --- | --- | --- | --- | --- | --- | --- | --- | --- | --- |
| **Medium** | **0** | 22 ± 5 | 97 ± 51 | 585 ± 261 | 112 ± 48 | 5 ± 1 | 7 ± 1 | 92 ± 15 | 13 ± 2 | 113 ± 31 |
| **HAC1** | **0.1** | 66 ± 20 | 254 ± 124 | 776 ± 428 | 145 ± 74 | 5 ± 1 | 7 ± 1 | 85 ± 17 | 12 ± 2 | 112 ± 32 |
|  | **1** | 124 ± 38 | 528 ± 249 | 594 ± 411 | 107 ± 29 | 6 ± 1 | 9 ± 1 | 116 ± 24 | 13 ± 2 | 112 ± 29 |
|  | **10** | 160 ± 75 | 602 ± 307 | 579 ± 367 | 125 ± 60 | 6 ± 1 | 7 ± 1 | 83 ± 17 | 13 ± 2 | 121 ± 37 |
|  | **100** | 152 ± 49 | 564 ± 239 | 911 ± 469 | 150 ± 61 | 6 ± 1 | 9 ± 1 | 107 ± 23 | 13 ± 2 | 171 ± 54 |
| **SiO_2_** | **10** | 40 ± 12 | 178 ± 117 | 2184 ± 1067 | 146 ± 56 | 7 ± 1 | 8 ± 1 | 87 ± 16 | 14 ± 2 | 190 ± 44 |
|  | **100** | 29 ± 3 | 323 ± 171 | 6933 ± 1535 | 211 ± 54 | 8 ± 1 | 16 ± 2 | 298 ± 152 | 17 ± 3 | 399 ± 65 |
|  | **1000** | 31 ± 4 | 391 ± 141 | 10996 ± 15445 | 269 ± 56 | 10 ± 1 | 18 ± 3 | 471 ± 347 | 21 ± 3 | 106 ± 18 |
|  | **10000** | 21 ± 3 | 80 ± 13 | 2862 ± 647 | 396 ± 51 | 6 ± 1 | 14 ± 2 | 127 ± 21 | 15 ± 2 | 23 ± 3 |
| **HAC1-NP** | **0.1 + 10** | 82 ± 18 | 225 ± 87 | 1662 ± 853 | 130 ± 54 | 6 ± 1 | 9 ± 1 | 114 ± 18 | 15 ± 2 | 125 ± 31 |
|  | **1 + 100** | 214 ± 90 | 823 ± 376 | 6846 ± 1291 | 213 ± 57 | 9 ± 1 | 15 ± 2 | 137 ± 23 | 18 ± 3 | 610 ± 246 |
|  | **10 + 1000** | 245 ± 88 | 1226 ± 535 | 10674 ± 1423 | 232 ± 39 | 9 ± 1 | 14 ± 2 | 128 ± 20 | 20 ± 3 | 124 ± 20 |
|  | **100 + 10000** | 68 ± 16 | 356 ± 138 | 4500 ± 963 | 354 ± 49 | 6 ± 1 | 10 ± 1 | 120 ± 20 | 14 ± 2 | 32 ± 9 |
| **LPS** | **0.1** | 24 ± 4 | 889 ± 528 | 3707 ± 721 | 5477 ± 3066 | 8 ± 1 | 16 ± 2 | 306 ± 177 | 19 ± 3 | 1211 ± 253 |
